# Supplementary material for: 3D multiparametric ultrasound imaging of steatotic liver disease in a study with male rats
Source: Nat Commun. 2025 Nov 20;16:10226. doi: 10.1038/s41467-025-65046-x (PMC12635210; doi:10.1038/s41467-025-65046-x)
Supplement: Supplementary file 1 — Supplementary Information [file 41467_2025_65046_MOESM1_ESM.pdf]

Supplementary Information for "3D multiparametric ultrasound imaging of steatotic liver disease in a study with male rats"

Donghyun Lee[1,2†], Jinseok Heo[2,3†], Hyeonji Mun[2,4†], Donghyeon Oh[1,2*], Yongjoo Ahn[1,2,4*], and Chulhong Kim[1,2,3,4,5,6*]

[1]Departments of Convergence IT Engineering, Pohang University of Science and Technology (POSTECH), Pohang, Republic of Korea

[2]Medical Device Innovation Center, Pohang University of Science and Technology (POSTECH), Pohang, Republic of Korea

[3]Departments of Electrical Engineering, Pohang University of Science and Technology (POSTECH), Pohang, Republic of Korea

[4]Departments of Medical Science and Engineering, Pohang University of Science and Technology (POSTECH), Pohang, Republic of Korea

[5]Departments of Mechanical Engineering, Pohang University of Science and Technology (POSTECH), Pohang, Republic of Korea

[6]Opticho Inc., Pohang, Republic of Korea

†These authors contributed equally to this work

*Corresponding authors: Chulhong Kim (chulhong@postech.ac.kr), YongJoo Ahn (ahnyj@postech.ac.kr), Donghyeon Oh (lumin9219@postech.ac.kr)

Table of Contents

**Supplementary Note 1**	Methodological criteria for scanning step size selection
**Supplementary Note 2**	Justification of sample size
**Supplementary Figure 1**	11-angle planewave US transmission scheme
**Supplementary Figure 2**	UFD processing
**Supplementary Figure 3**	Vascular modeling
**Supplementary Figure 4**	Volumetric 3D UFD in other liver lobes
**Supplementary Figure 5**	Animal experimental schemes for the SLD progression monitoring and validation of hepatic vasculature alterations in SLD
**Supplementary Figure 6**	Representative 3D and 2D PD images

	acquired from normal and SLD-conditioned groups at weeks 0, 2, 4, 6, and 8
**Supplementary Figure 7**	Changes in weight during the 8-week monitoring period, and changes from the original VVO and FMBV values
**Supplementary Figure 8**	Oil Red O (ORO), CD31, and hematoxylin and eosin (H&E) staining results, ordered by steatosis grades
**Supplementary Figure 9**	LYVE1 and CD 31 staining results from the SLD progression monitoring and validation schemes at SLD-induced weeks 0, 2, 4, and 8
**Supplementary Figure 10**	Animal experimental schemes for SLD progression and recovery monitoring
**Supplementary Figure 11**	Representative 3D and 2D PD images acquired from the recovery group at weeks 0, 2, 4, 6, and 8
**Supplementary Figure 12**	LYVE1 and CD 31 quantification results from the SLD progression and recovery monitoring and validation schemes at SLD-induced weeks 0, 2, 4, and 8.
**Supplementary Figure 13**	ROC curve analyses of 3D USI indices
**Supplementary Figure 14**	Correlation and ROC curve analyses of 2D UFD indices
**Supplementary Figure 15**	Variability of 2D UFD indices
**Supplementary Figure 16**	Performance of nested Monte Carlo 5-fold cross-validation based on combinations of indices
**Supplementary Figure 17**	Inter-subject reproducibility of UFD indices in the steady-state rats (n = 17, at week 0)
**Supplementary Figure 18**	Results of inter-system agreement experiments using UFD

**Supplementary Figure 19**	Human liver UFD image using GE C1-6-D probe.
**Supplementary Table 1**	List of acronyms
**Supplementary Table 2**	Statistical analyses of the SLD progression monitoring experiments
**Supplementary Table 3**	Histological quantification results of the SLD progression monitoring and validation experiments
**Supplementary Table 3**	Statistical analyses of the SLD recovery monitoring experiments
**Supplementary Table 4**	Comparative histological quantification results between SLD recovery and SLD progression monitoring with validation experiments
**Supplementary Table 6**	Combination order of all US indices
**Supplementary Video 1**	Real-time respiratory triggering and ensemble selection
**Supplementary Video 2**	3D hepatic USI sequence, liver region segmentation, and quantitative 3D PD through vascular modeling

Supplementary Note 1

Methodological criteria for scanning step size selection

A dense 3D volumetric scan step size was selected to enhance visualization of vascular network connectivity and to enable more precise implementation of vascular morphological indices. Although the transducer specifications indicate an elevational width of 1.6 mm, the actual width of the ultrasound beam differs from this nominal value. According to the Rayleigh approximation[1], a mathematical model for measuring the elevational beam width of a transducer, the elevational beam width is approximately equal to (c/f)*F/H, where c is speed of sound, f is the US frequency, F is the elevation focus, and H is the elevation width. In our case, c = 1500 m/s (the sound speed at which macrovascular structures were optimally visualized in SLD weeks 3–4 B-mode liver imaging), f = 15.625 MHz (carrier frequency), F = 8 mm, and H = 1.6 mm. Therefore, the theoretically estimated elevational beam width is approximately 0.48 mm, which is consistent with the value reported in the literature[2]. Accordingly, considering spatial sampling based on the Nyquist sampling theorem, a minimum elevational step size of 0.24 mm (half the beam width) is required to precisely reconstruct signals at spatial locations. Taking into account the pixel size (0.05 mm) in our 2D imaging setup and the need for acquisition efficiency, an elevational step size of 0.2 mm was selected.

Supplementary Note 2

Justification of sample size

We have three experimental schemes: (1) an SLD progression monitoring scheme involving normal and SLD-conditioned rats, (2) an SLD validation scheme using groups of rats divided based on induced-SLD durations, and (3) an SLD recovery monitoring scheme for recovery-conditioned rats. The sample sizes for these schemes were determined using G*Power[3, 4], based on previous research findings that hepatic fat accumulation increased proportionally with the duration of MCD diet induction, reaching approximately 40% at six weeks[5]. Compliance with the 3R principles (Replacement, Reduction, and Refinement) was also carefully considered.

(1) For the SLD progression monitoring scheme, which aimed to compare group-specific trends over time, an a priori power analysis was performed to estimate the required sample size using an F-test based on repeated-measures ANOVA (within-between interaction). The input parameters were set as follows: the effect size, f, was set to 0.4, based on previous studies showing that fat accumulation increased steadily over time and reached approximately 40% at week 6, indicating a large effect size. The significant level (α, labeled as "α error prob" in G*Power) and statistical power ($1 - \beta$, labeled as "power ($1 - \beta$ err prob)" in G*Power) were set to commonly used values of 0.05 and 0.80, respectively. The number of groups and number of measurements were set to 2 and 9, respectively, according to the 8-week monitoring design. The correlation among repeated measures and the nonsphericity correction, ε, were both conservatively set to 0.5. Based on these parameters, the required sample size was calculated to be 8, resulting in a sample size of n = 4 for each group (Normal and SLD).

(2) Regarding the SLD validation scheme, we sought to examine the correlation between fat percentage and CD31 density across weeks. Therefore, an a priori power analysis was conducted employing an exact test for correlation under the bivariate normal model. The input parameters were set as follows: The number of tails was set to two for two-tailed testing. The correlation coefficient under the alternative hypothesis (ρ_1, labeled as "Correlation ρ H1" in G*Power) was assumed to be 0.6, reflecting the moderately strong correlation between fat percentage and CD31 density. The significant level and statistical power were set to the commonly used values of 0.05 and 0.80, respectively. The correlation under the null hypothesis (ρ_0, labeled as "Correlation ρ H0" in G*Power) was set to 0, the typical assumption for the null hypothesis. Based on these parameters, the calculated sample size was 19; however, considering the potential uncertainty in the correlation, the sample size was conservatively set to n = 25. The sample size of n = 25 was distributed across five groups (weeks 0, 1, 2, 3, and 4) to ensure clear identification of early vascular changes associated with progressive fat accumulation.

(3) In the SLD recovery monitoring scheme, the sample size was set to n = 4, consistent with the SLD progression monitoring scheme described in (1). Consequently, the total sample size was n = 37.

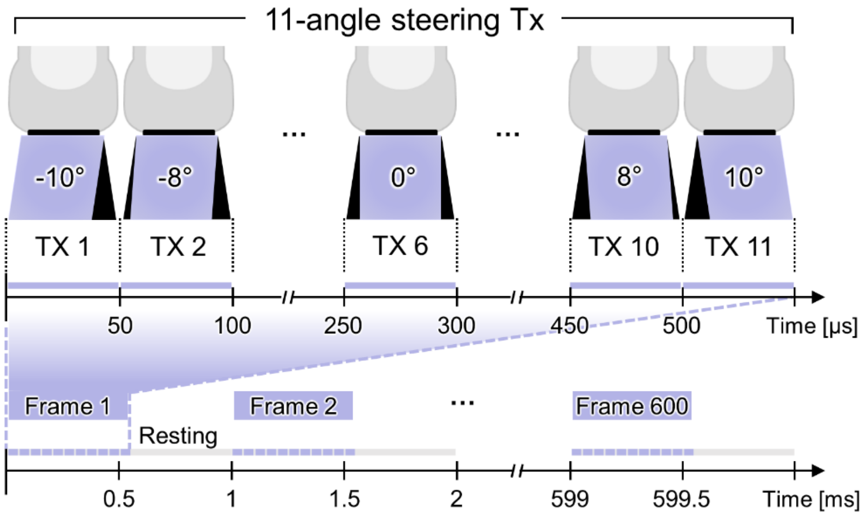

Supplementary Fig. 1. 11-angle planewave US transmission scheme.

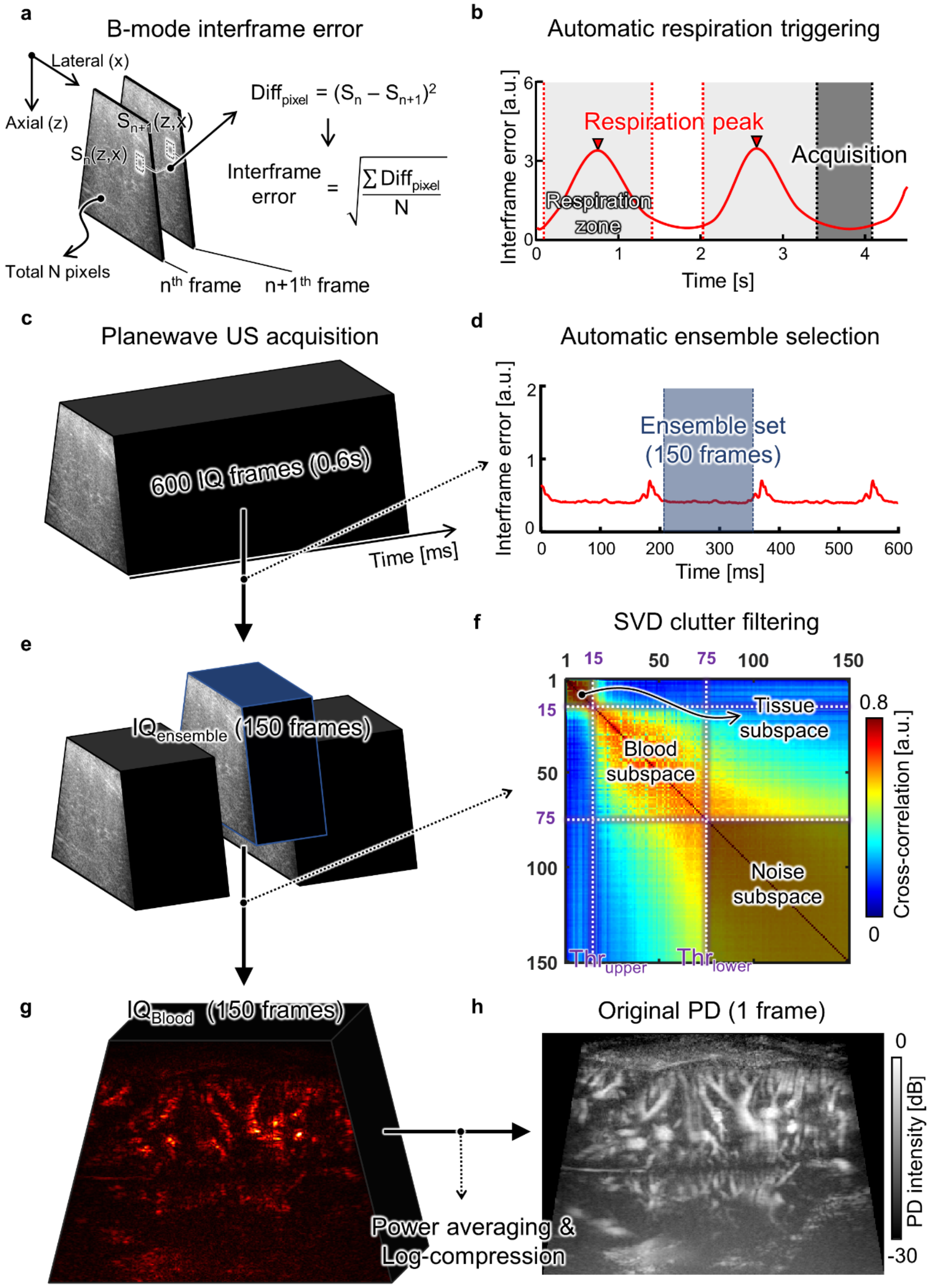

Supplementary Fig. 2. UFD processing. **a**, Root mean square (RMS) calculation-based US B-mode interframe error. **b**, Automatic respiration triggering with a frame rate of 20Hz, applying a moving average. **c**, Planewave US acquisition at a single imaging location. **d**, Automatic ensemble selection through least difference in interframe errors. **e**, Selected IQ ensemble frames. **f**, Spatiotemporal singular value decomposition (SVD)-based clutter filtering. **g**, Filtered blood signals. **h**, Original PD image through power averaging and logarithmic compression.

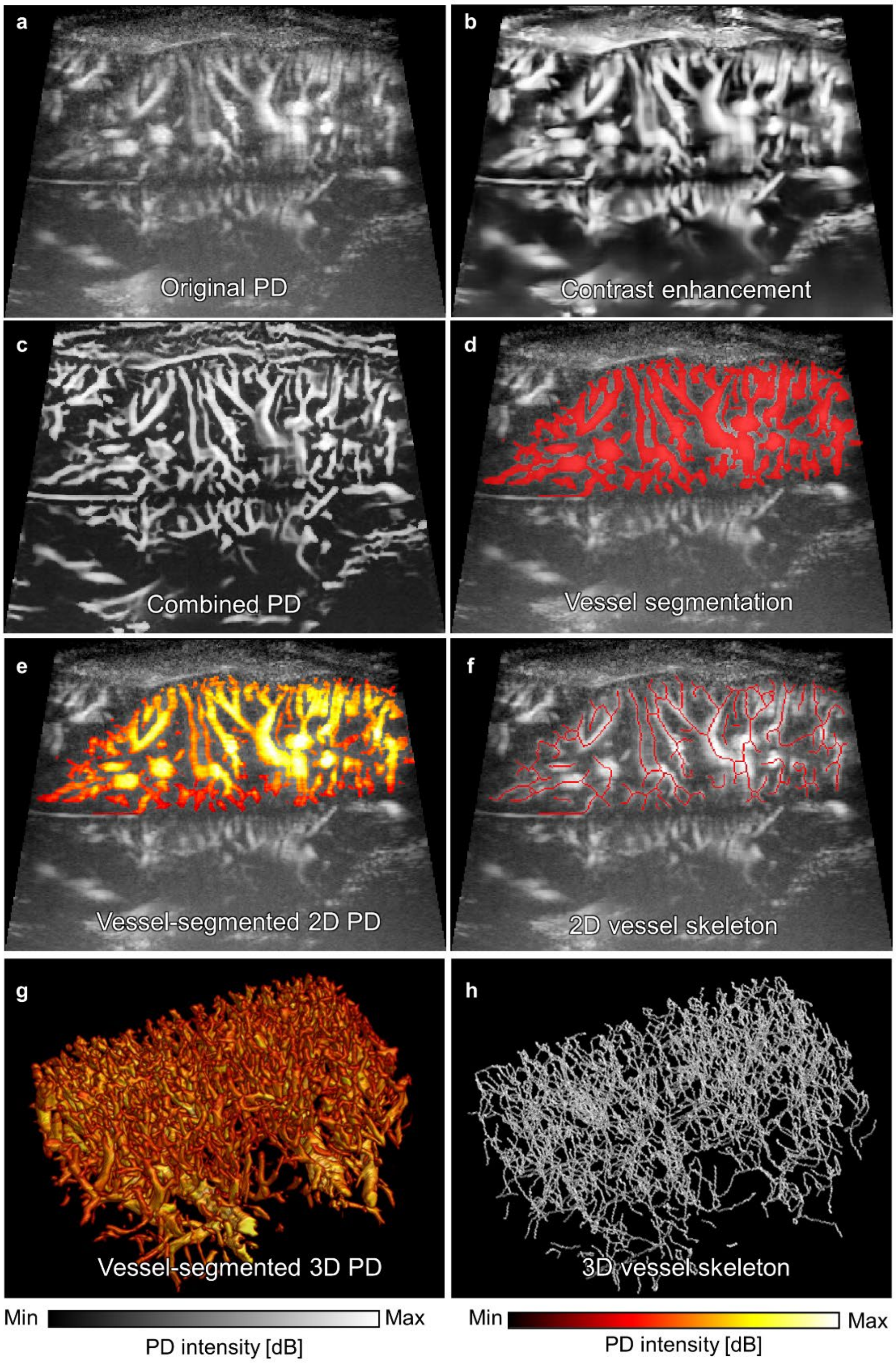

Supplementary Fig. 3. Vascular modeling. a, Original PD image obtained from UFD processing. **b**, Contrast enhanced image processed by removing inherent noise, reducing speckle effects, and applying intensity equalization. **c**, Composed image generated by combining the PD image with the Jerman-filtered image. **d**, Vessel mask generated by thresholding the combined image. **e**, Vessel-segmented 2D PD image. **f**, 2D vessel skeleton generated by applying the skeletonization process to the 2D vessel mask. **g**, Vessel-segmented 3D PD image. **h**, 3D vessel skeleton generated by applying the skeletonization process to the 3D vessel mask.

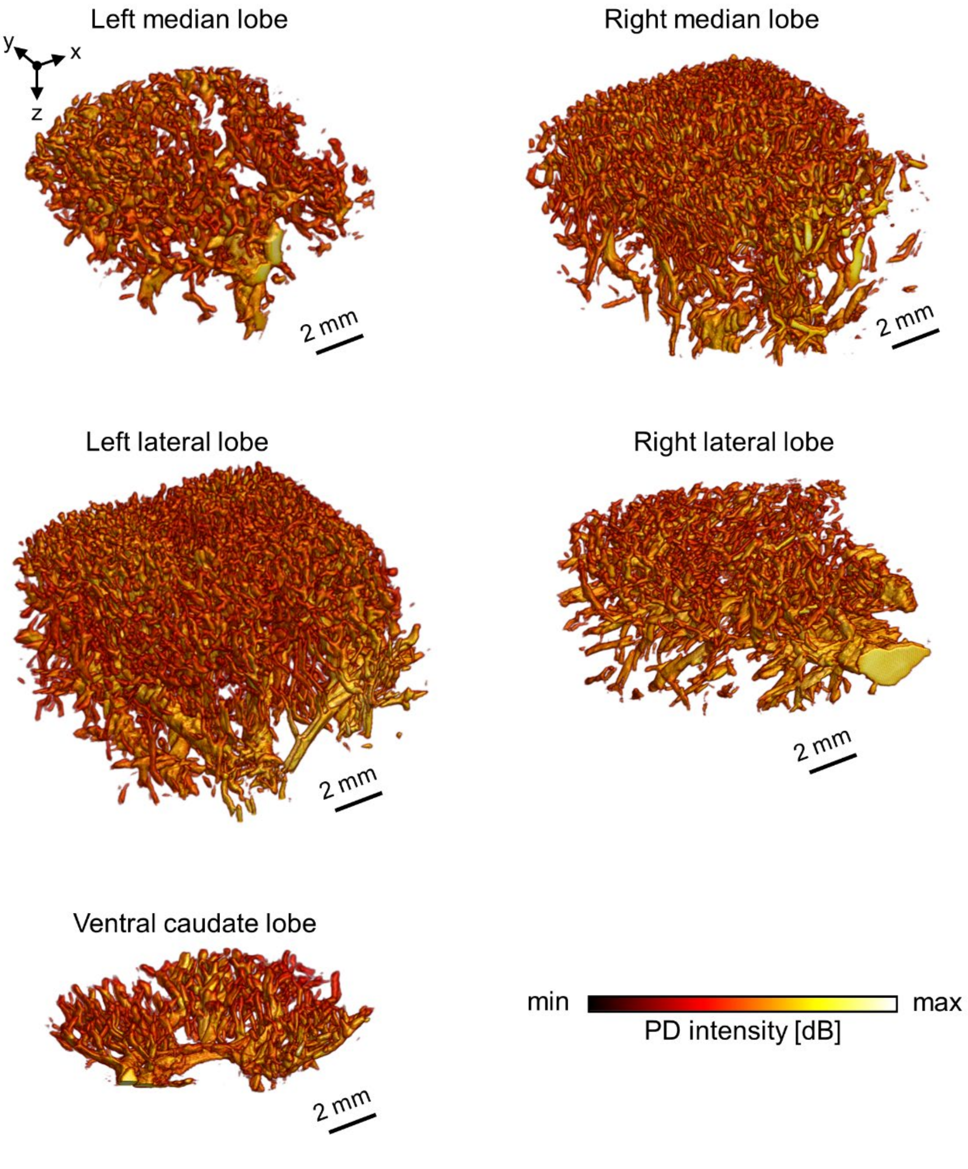

Supplementary Fig. 4. Volumetric UFD in other liver lobes.

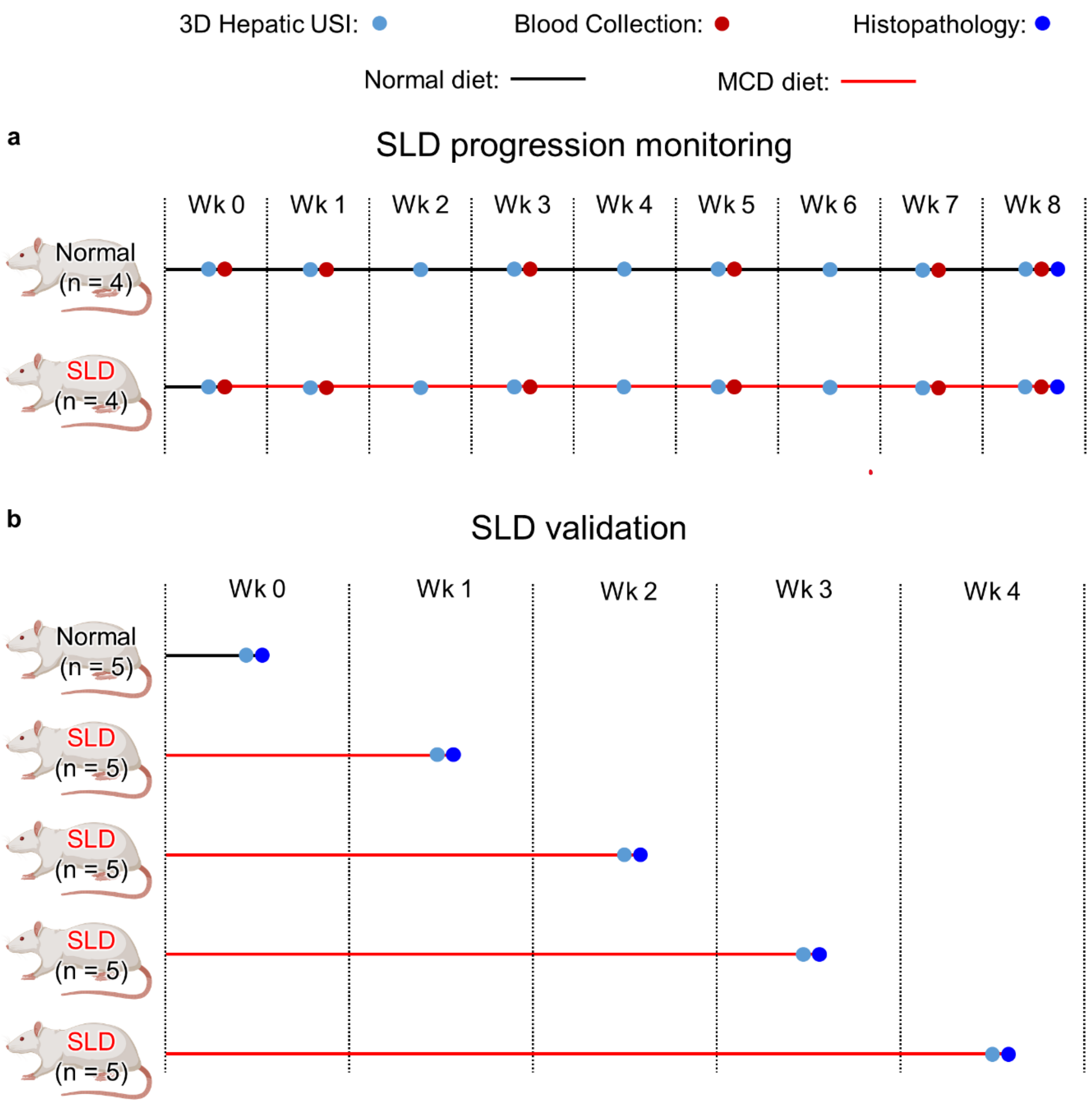

Supplementary Fig. 5. Animal experimental schemes for the SLD progression monitoring and validation of hepatic vasculature alterations in SLD. **a**, SLD progression monitoring scheme. **b**, SLD validation scheme. MCD, methionine-choline deficient and SLD, steatotic liver disease. Rat schematic in panel (a) and (b) was created in BioRender. Ahn, M. (2025) https://BioRender.com/yz7h2m4.

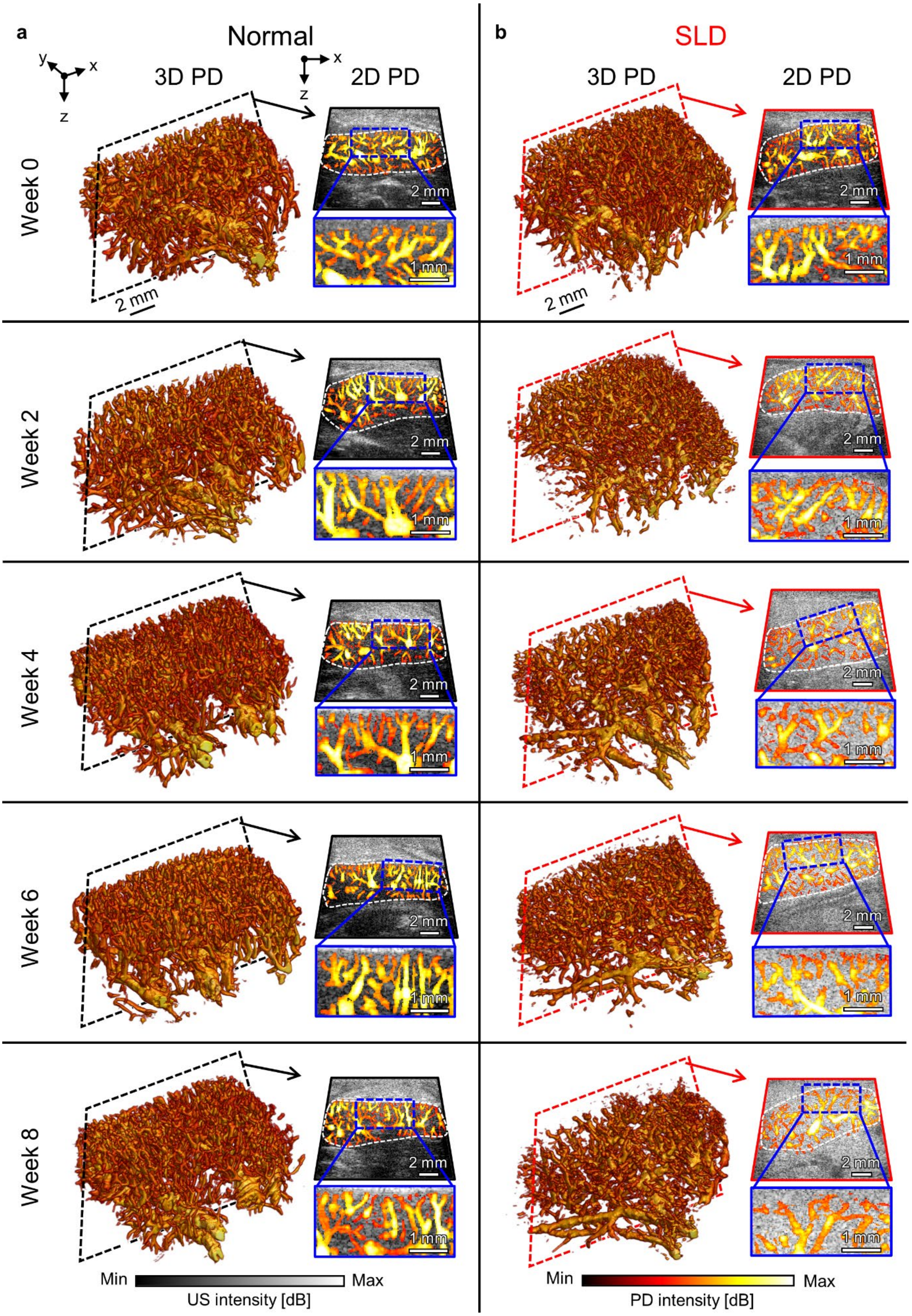

Supplementary Fig. 6. Representative 3D and 2D PD images acquired from normal and SLD-conditioned groups at weeks 0, 2, 4, 6, and 8. a, Normal group. **b**, SLD group.

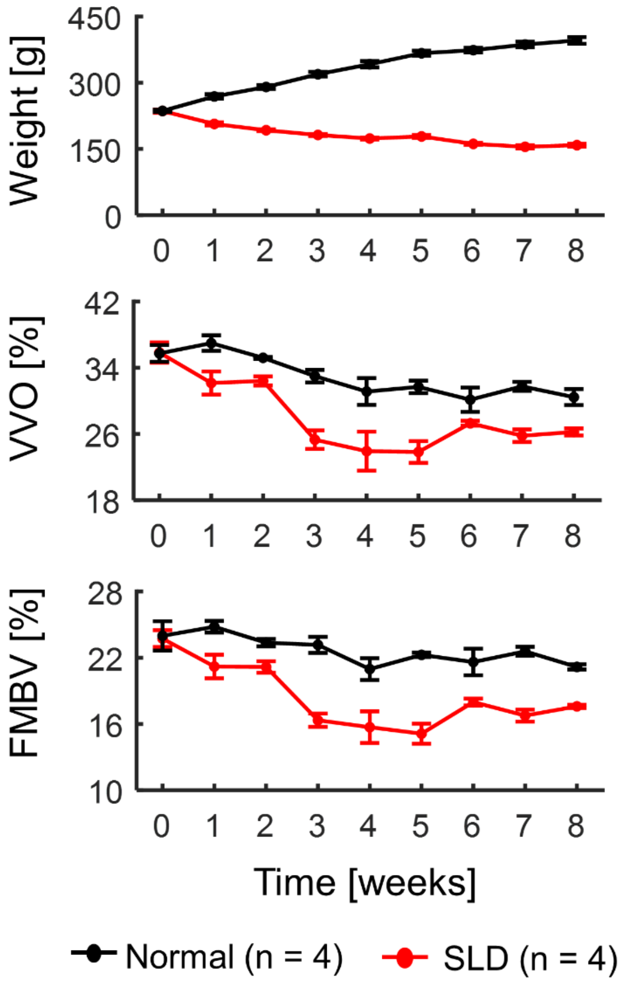

Supplementary Fig. 7. Changes in weight during the 8-week monitoring period, and changes from the original VVO and FMBV values. VVO, vessel volume occupancy; FMBV, fractional moving blood volume. All data are presented as mean ± SEM. Source data are provided as a Source Data file.

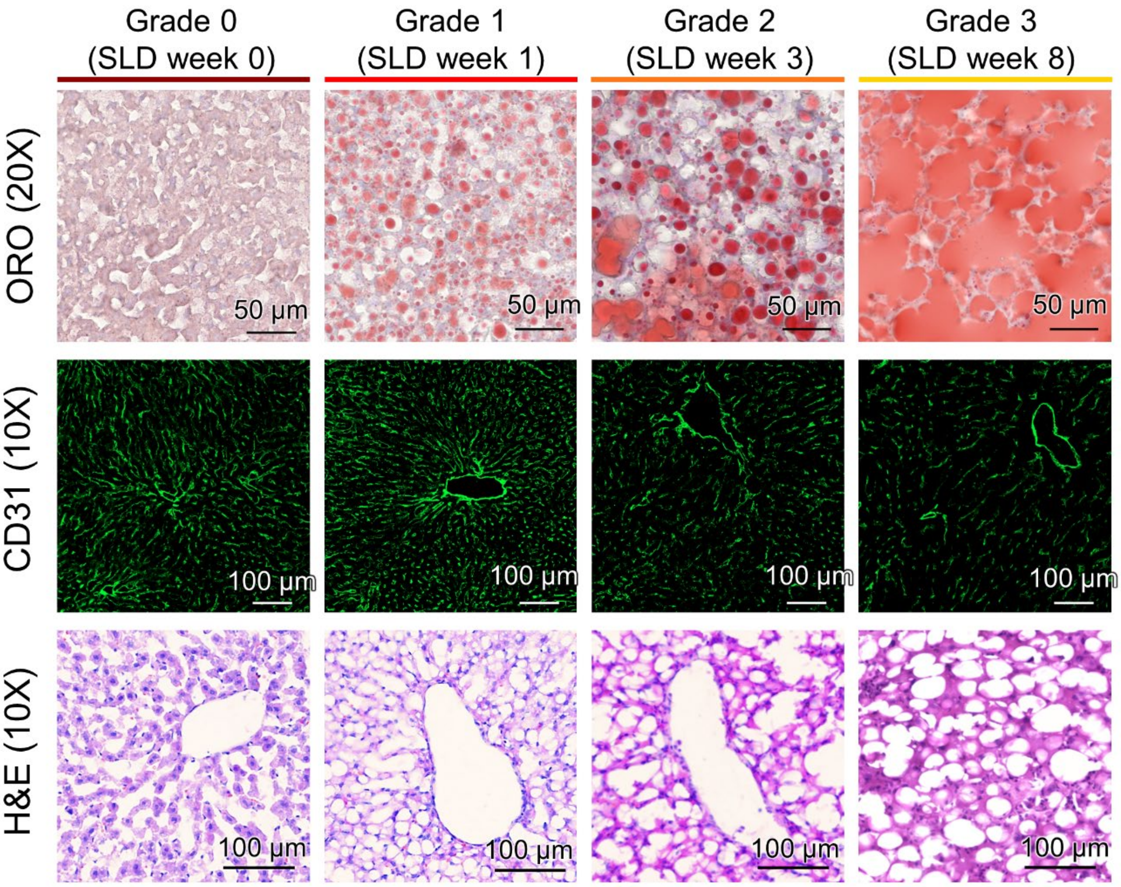

Supplementary Fig. 8. Oil Red O (ORO), CD31, and hematoxylin and eosin (H&E) staining results, ordered by steatosis grades. Microscopic staining images of excised tissue samples based on steatosis grades 0–3. Lipid droplets, micro-vascular wall structures rarefaction, and enlarged cellular structures are commonly observed in each stained microscope image of grades 1, 2, and 3. The severity of these histopathologic features is greater for higher grades. CD31 signals are shown in green.

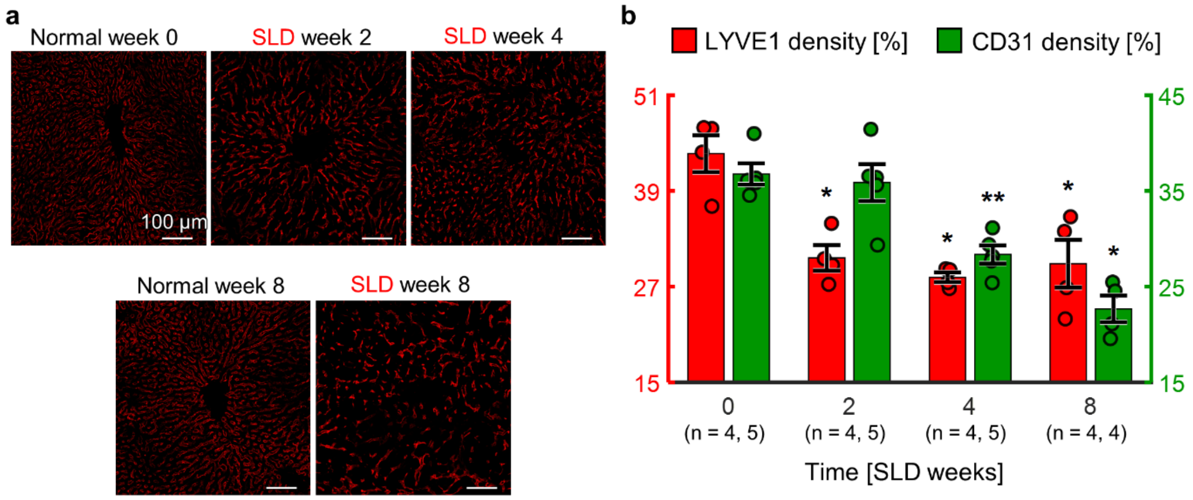

Supplementary Fig. 9. LYVE1 and CD31 staining results from the SLD progression monitoring and validation schemes at SLD-induced weeks 0, 2, 4, and 8. a, Representative microscopic LYVE1 staining images (red) from the Normal and SLD groups. **b**, Quantitative analysis of LYVE1 and CD31 densities in the Normal and SLD groups across SLD-induced weeks 0, 2, 4, and 8. Two-sided Mann–Whitney test without adjustment for multiple comparison was used to compare each time point (only comparisons with week 0 are shown; $*p < 0.05$, $**p < 0.01$, $***p < 0.001$). All statistical results, including other comparisons and exact p-values, are provided in Supplementary Table 3. All data are presented as mean ± SEM. Source data are provided as a Source Data file.

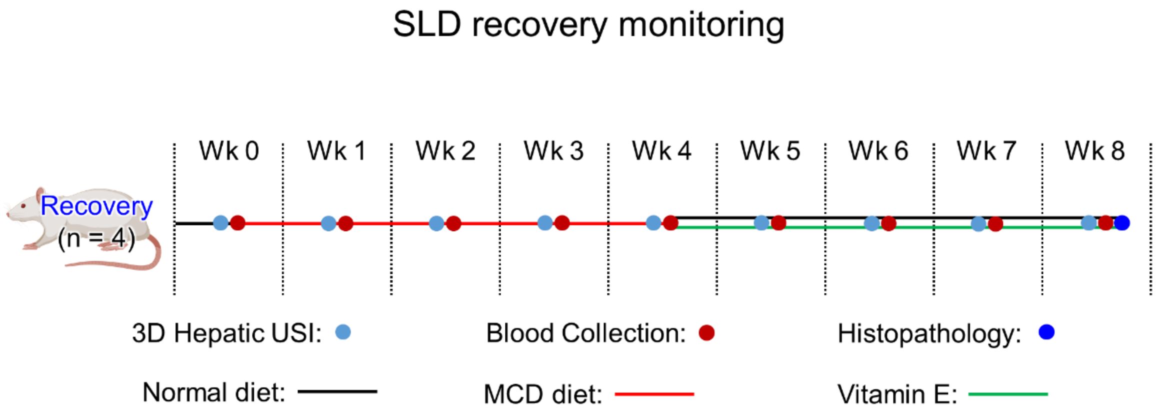

Supplementary Fig. 10. Animal experimental scheme for SLD progression and recovery monitoring. MCD, methionine-choline deficient; SLD, steatotic liver disease. Rat schematic was created in BioRender. Ahn, M. (2025) https://BioRender.com/yz7h2m4.

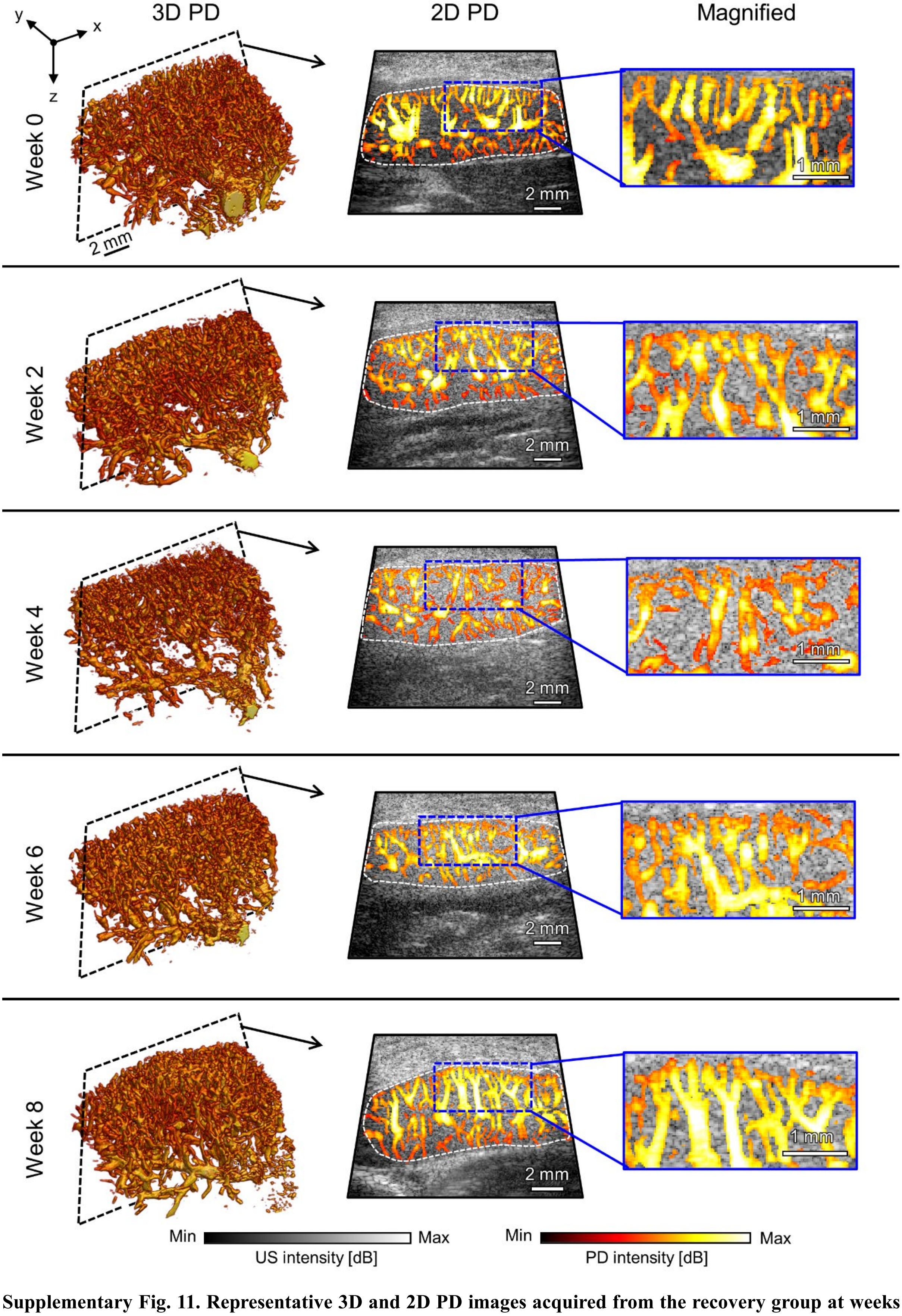

Supplementary Fig. 11. Representative 3D and 2D PD images acquired from the recovery group at weeks 0, 2, 4, 6, and 8.

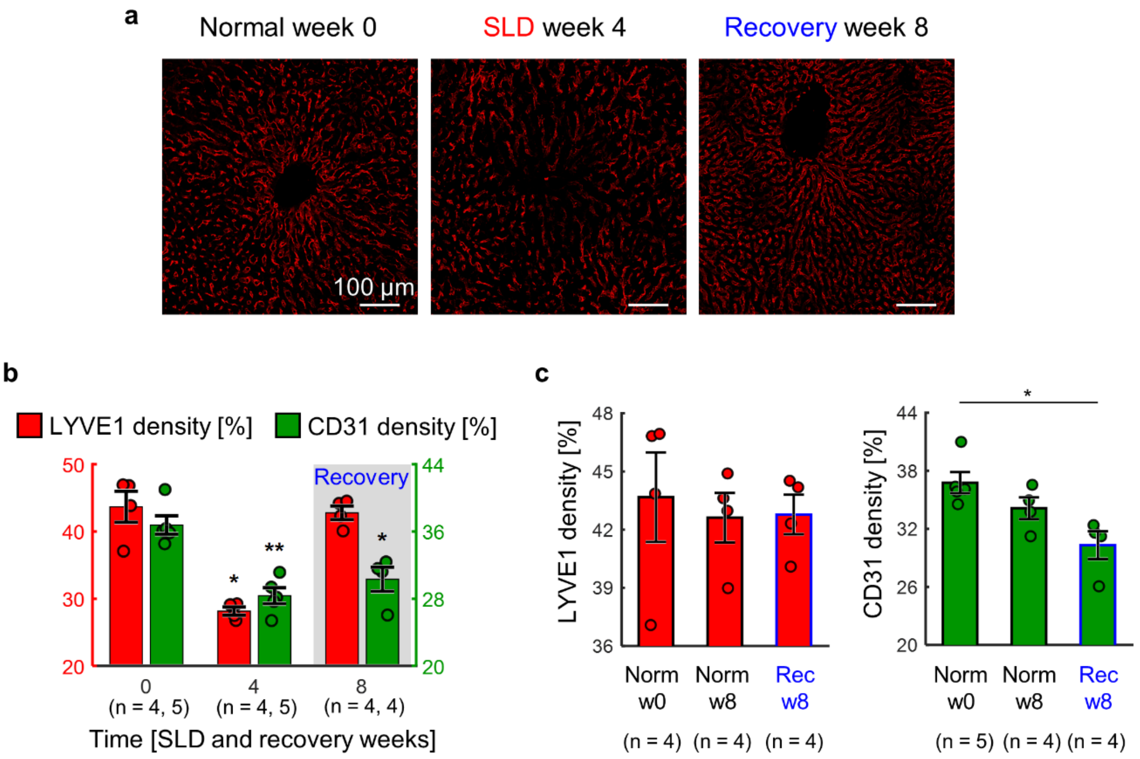

Supplementary Fig. 12. LYVE1 and CD 31 quantification results from the SLD progression and recovery monitoring and validation schemes at SLD-induced weeks 0, 2, 4, and 8. a, Representative LYVE1 staining images (red) from the SLD progression, validation, and recovery schemes. **b**, Temporal quantification trends of LYVE1 and CD31 densities during SLD progression and recovery. Two-sided Mann–Whitney test without adjustment for multiple comparisons was used to compare each time point (only comparisons with week 0 are shown; $*p < 0.05$, $**p < 0.01$). **c**, Comparison of LYVE1 and CD31 densities between the normal and SLD recovery states. Two-sided Mann–Whitney test without adjustment for multiple comparisons was used to compare each time point (only case with $*p < 0.05$ is shown). Norm, normal; and Rec, recovery. All statistical results, including other comparisons and exact p-values, are provided in Supplementary Tables 4 and 5. All data are presented as mean ± SEM. Source data are provided as a Source Data file.

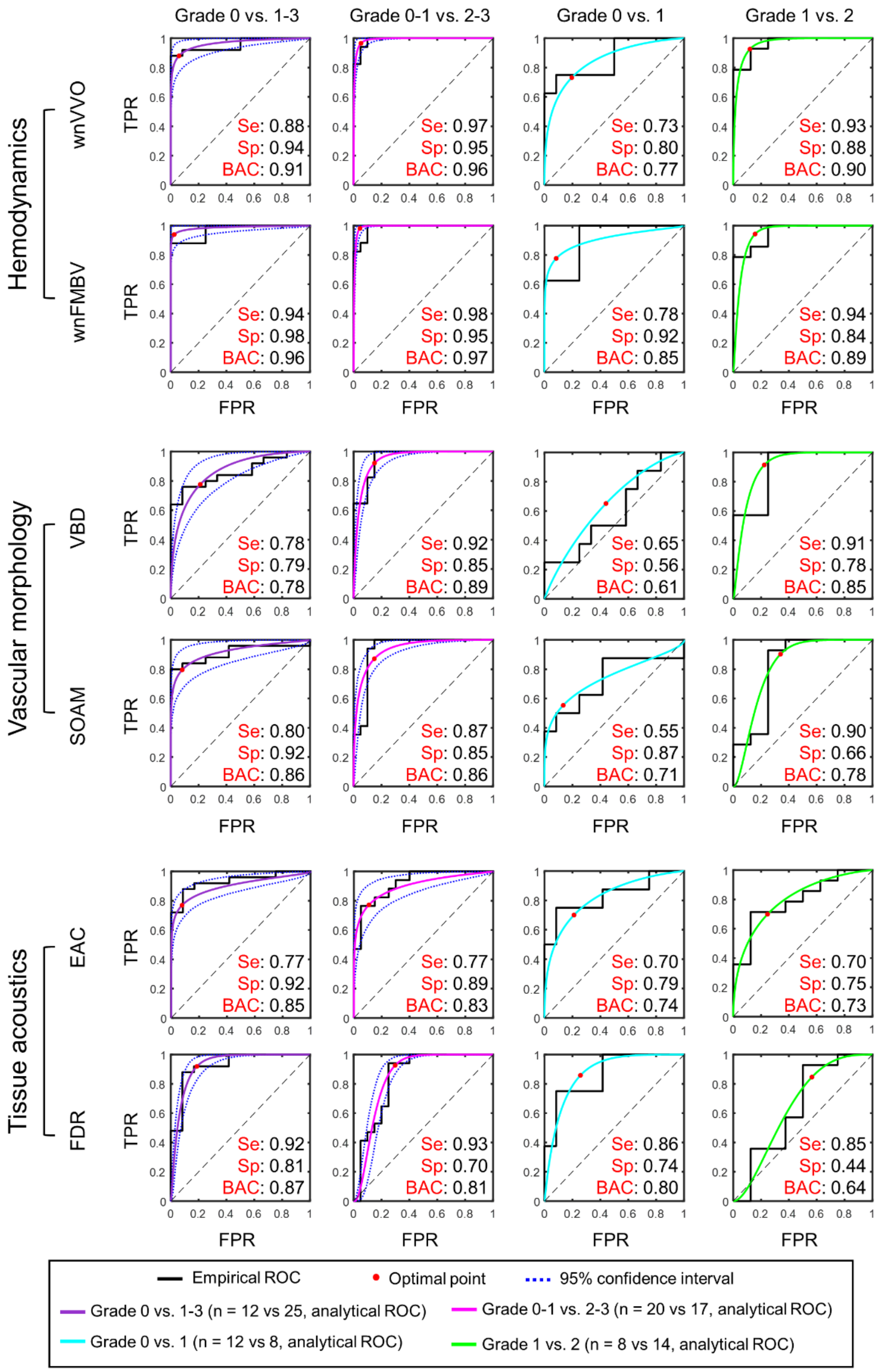

Supplementary Fig. 13. ROC curve analyses of 3D USI indices. UFD indices (weight-normalized vessel volume occupancy (wnVVO), weight-normalized fractional moving blood volume (wnFMBV), vessel bifurcation density (VBD), and sum of angles metric (SOAM)), ATI index (estimated attenuation coefficient (EAC)), and ASQ index (focal disturbance ratio (FDR)). In the classifications of grade 0 vs. 1 and grade 1 vs. 2, estimating 95% confidence intervals is excluded due to insufficient data. Se, sensitivity; Sp, specificity; and BAC, balanced accuracy. Source data are provided as a Source Data file.

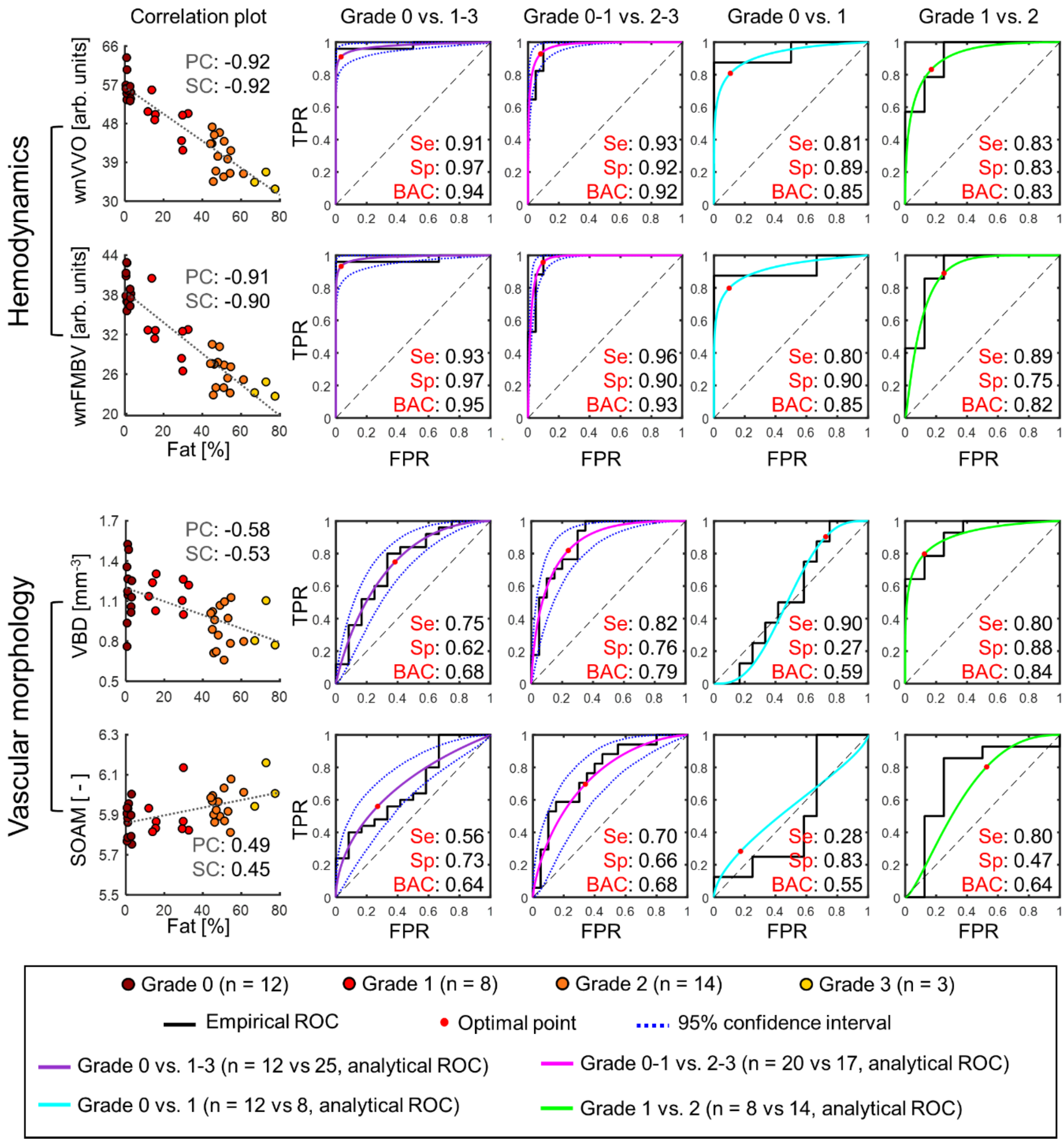

Supplementary Fig. 14. Correlation and ROC curve analyses of 2D UFD indices. Weight-normalized vessel volume occupancy (wnVVO), weight-normalized fractional moving blood volume (wnFMBV), vessel bifurcation density (VBD), and sum of angles metric (SOAM). In the classifications of grade 0 vs. 1 and grade 1 vs 2, estimating 95% confidence intervals is excluded due to insufficient data. Se, sensitivity; Sp, specificity; and BAC, balanced accuracy. Exact p-values are provided in source data Source data are provided as a Source Data file.

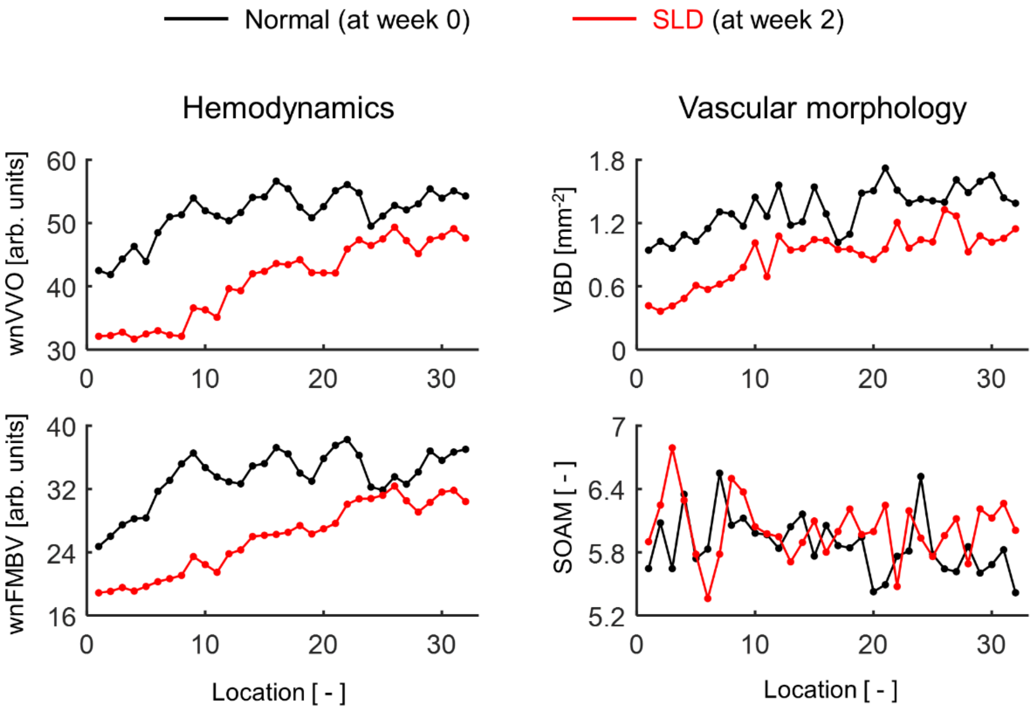

Supplementary Fig. 15. Variability of 2D UFD indices. Comparison of 2D UFD indices between normal rat at week 0 and SLD-conditioned rat at week 2. wnVVO, weight-normalized vessel volume occupancy; wnFMBV, weight-normalized fractional moving blood volume; VBD, vessel bifurcation density; and SOAM, sum of angles metric. Source data are provided as a Source Data file.

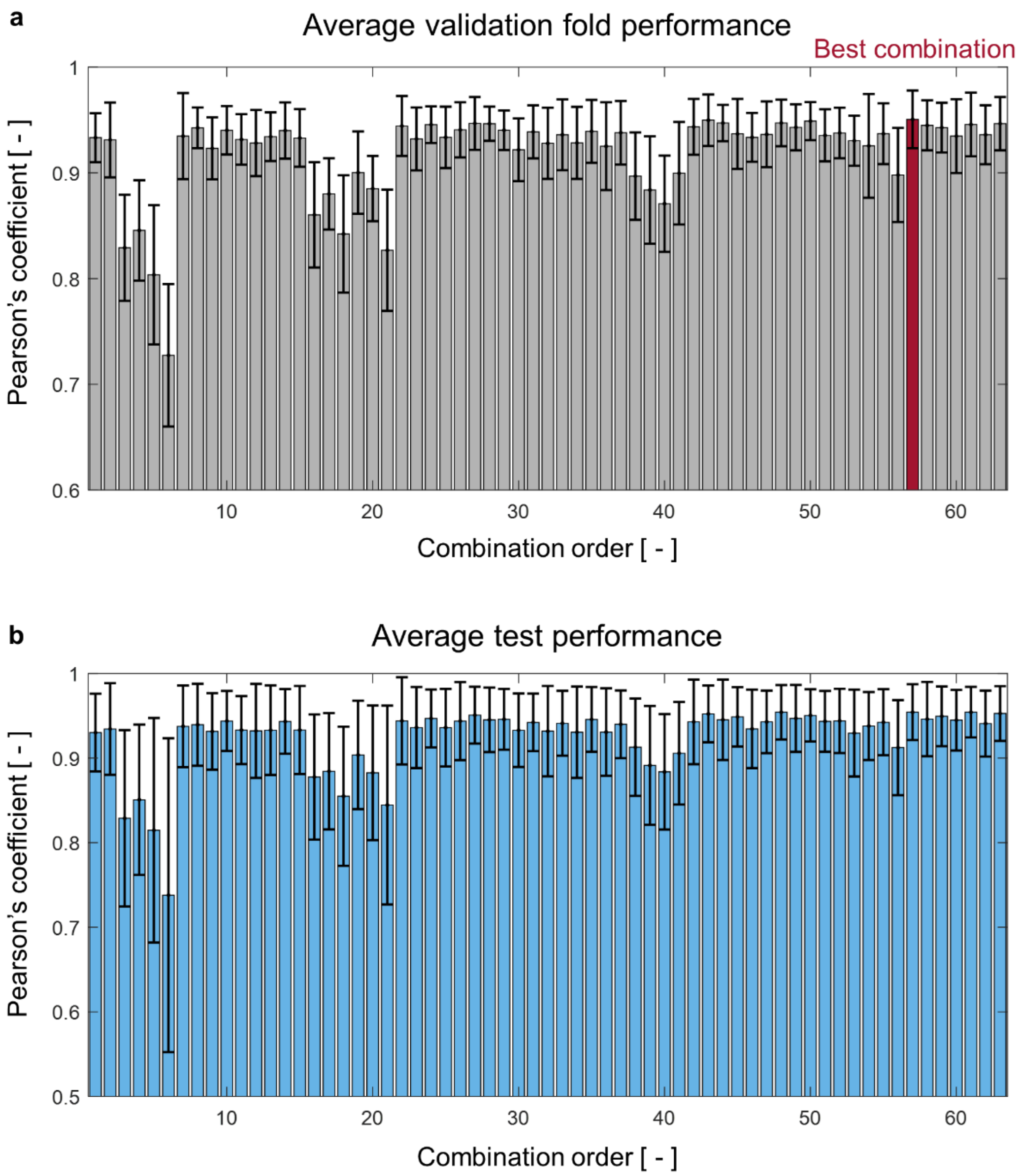

Supplementary Fig. 16. Performance of nested Monte Carlo 5-fold cross-validation based on combinations of indices. a, Validation fold performance used to select the optimal feature combination. **b**, Test set performance for evaluating generalization capability. The number of combined indices increases with the order of combinations. The performance of (a) and (b) was evaluated using the mean Pearson's coefficient over 100 trials for each combination order. Data are presented as mean ± SD. A list of combinations is provided in Supplementary Table 6. Source data are provided as a Source Data file.

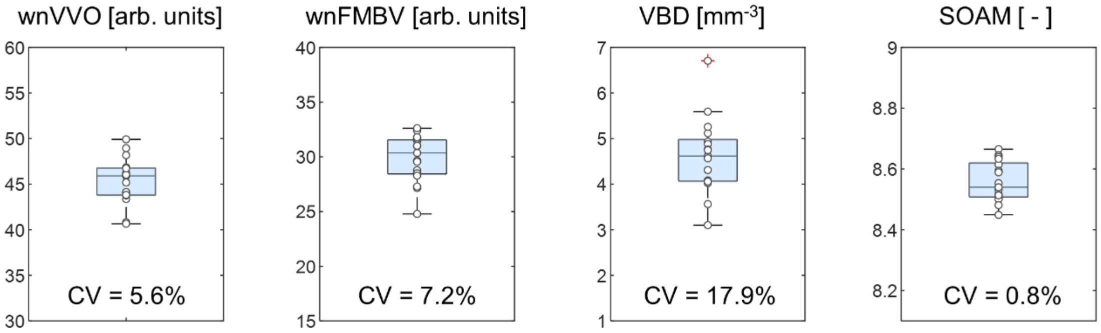

Supplementary Fig. 17. Inter-subject reproducibility of UFD indices in the steady-state rats (n = 17, at week 0). CV, coefficient of variation. In each boxplot, the center line indicates the median value, the box edges represent the first and third quartiles, and the whiskers extend to the minimum and maximum values. Source data are provided as a Source Data file.

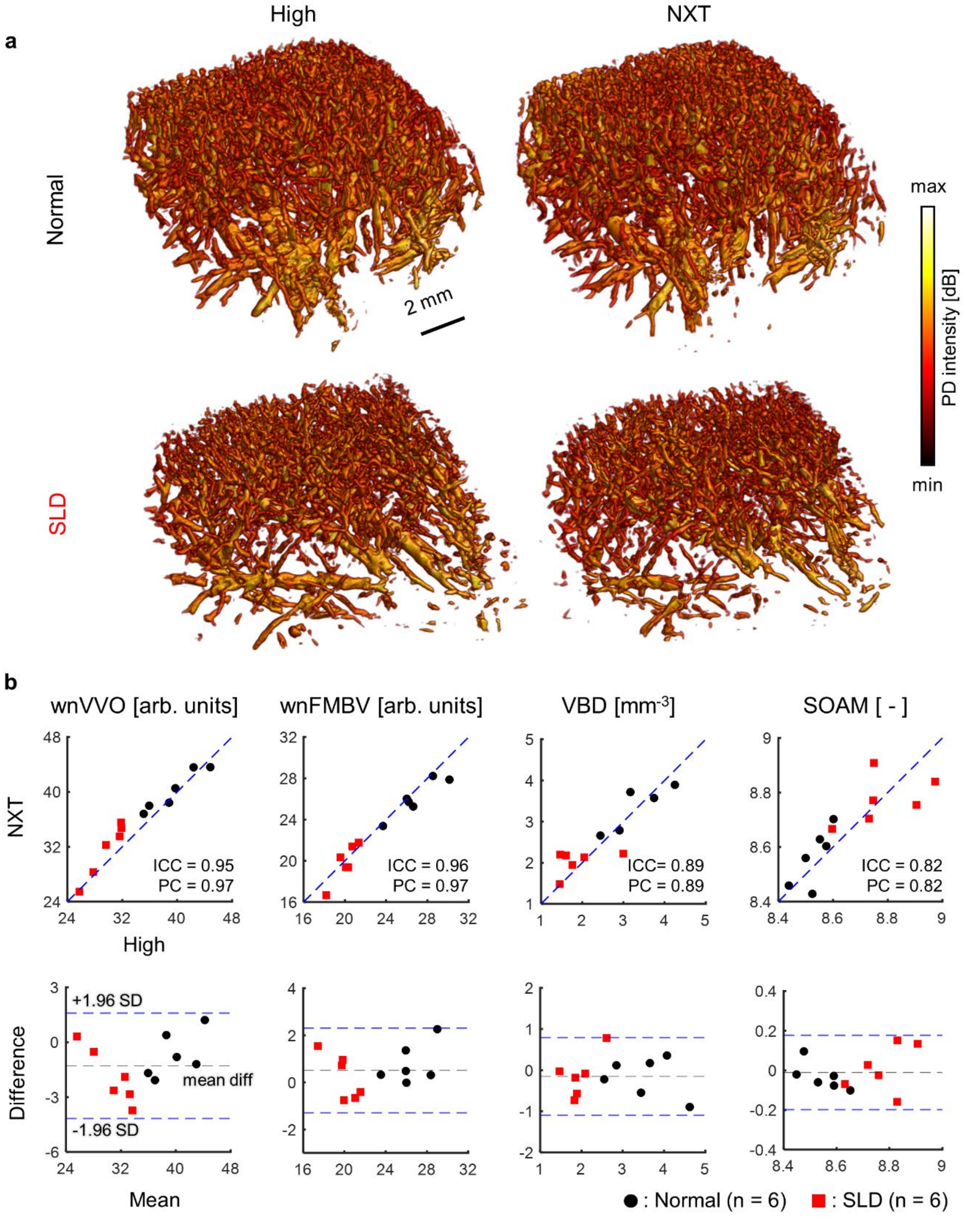

Supplementary Fig. 18. Results of inter-system agreement experiments using UFD. a, Volumetric PD images acquired using Vantage High and NXT systems for Normal and SLD rats at week 4. **b**, Scatter and Bland-Altman plots comparing the UFD quantification results, including the intraclass correlation coefficient and Pearson's coefficient. SLD, steatotic liver disease; wnVVO, weight-normalized vessel volume occupancy; wnFMBV, weight-normalized fractional moving blood volume; VBD, vessel bifurcation density; SOAM, sum of angles metric; ICC, intraclass correlation coefficient; PC, Pearson's correlation coefficient; SD, standard deviation, and diff, difference. Source data are provided as a Source Data file.

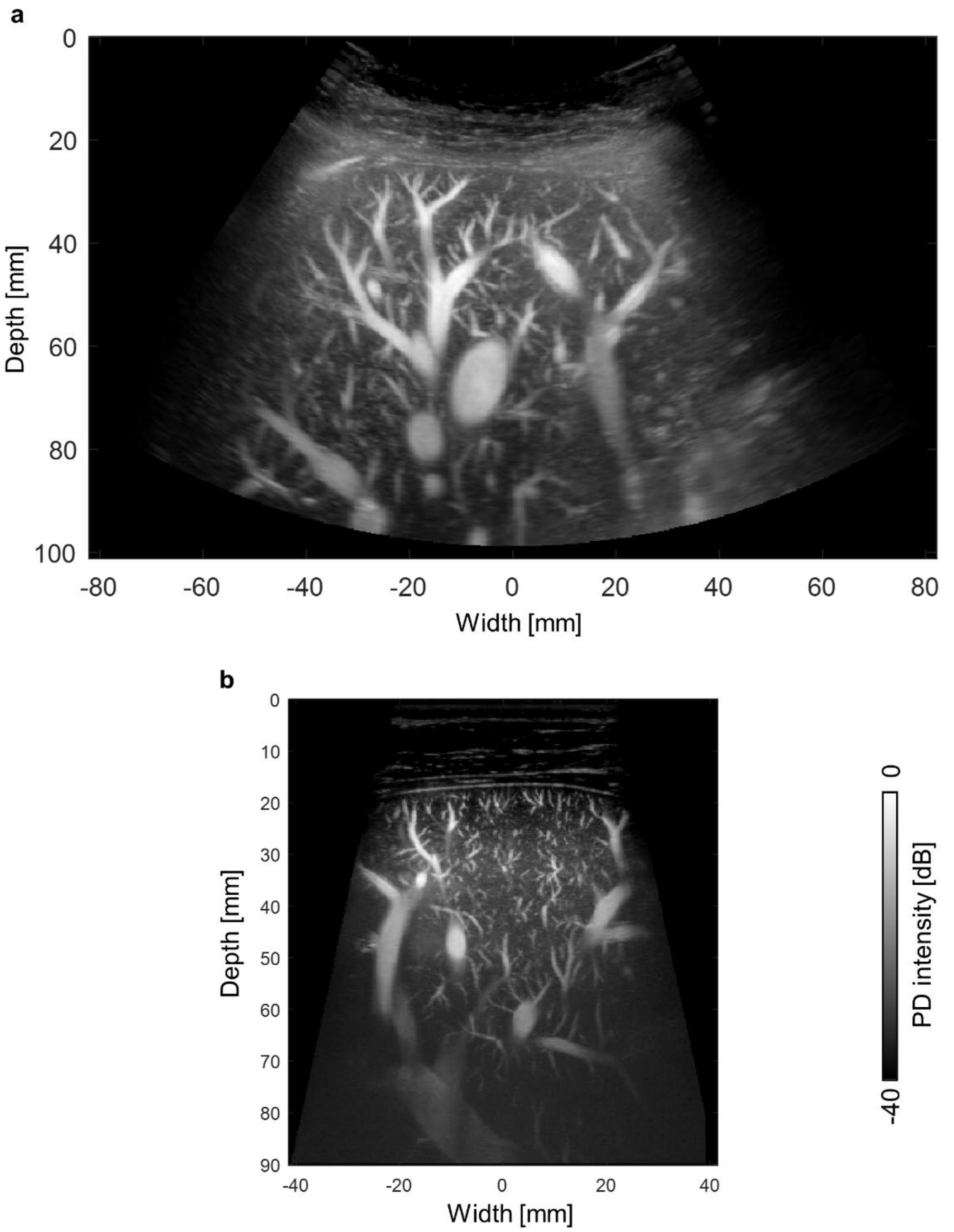

Supplementary Fig. 19. Human liver UFD images from a healthy volunteer (male, 20–30 years old). a, Cross-sectional PD image obtained using convex-array US probe (GE C1-6-D). **b**, Cross-sectional PD image obtained using linear-array US probe (GE 9L-D). **c**, Magnified views of (a) and (b). Vantage 256 system was employed for imaging. 400 frames (500 Hz frame rate) and 240 frames (300 Hz frame rate) were acquired via the intercostal window using convex-array and linear-array probes, respectively.

Supplementary Table 1. List of acronyms.

Acronyms	Paraphrase
SLD	Steatotic liver disease
USI	Ultrasound imaging
UFD	Ultrafast Doppler imaging
MRI	Magnetic resonance imaging
ATI	Attenuation imaging
ASQ	Acoustic structure quantification
SVR	Support vector regression
DAQ	Data acquisition
IQ	In-phase/quadrature
PD	Power Doppler
VVO	Vessel volume occupancy
FMBV	Fractional moving blood volume
VBD	Vessel bifurcation density
SOAM	Sum of angles metric
ROI	Region of interest
EAC	Estimated attenuation coefficient
FDR	Focal disturbance ratio
RML	Right median lobe
LML	Left median lobe
LLL	Left lateral lobe
VCL	Ventral caudate lobe

Acronyms	Paraphrase
HAs	Hepatic arteries
PVs	Portal veins
HVs	Hepatic veins
MHVs	Major hepatic vessels
PLVs	Peri-lobular vessels
ILAVs	Interlobular arteries and veins
SLVs	Sublobular veins
MCD	Methionine-choline deficient
ALT	Alanine aminotransferase
AST	Aspartate transaminase
TBIL	Total bilirubin
ORO	Oil Red O
H&E	Hematoxylin and eosin
wnVVO	Weight-normalized VVO
wnFMBV	Weight-normalized FMBV
PC	Pearson’s coefficient
SC	Spearman’s coefficient
ROC	Receiver operating characteristic
RF	Radiofrequency
SVD	Singular value decomposition
sROIs	Secondary ROIs

Supplementary Table 2. Statistical analyses of the SLD progression monitoring experiments. Quantitative US indices were monitored over 8 weeks. Weight-normalized vessel volume occupancy, wnVVO; weight-normalized fractional moving blood volume, wnFMBV; vessel bifurcation density, VBD; sum of angles metric, SOAM; estimated attenuation coefficient, EAC; and focal disturbance ratio, FDR. Blood biomarkers such as alanine aminotransferase (ALT), aspartate transaminase (AST), and total bilirubin (TBIL) were tested. Statistical significance was assessed using two-sided Mann–Whitney test without adjustment for multiple comparisons; *p < 0.05.

US index	Group	Week 0	Week 1	Week 2	Week 3	Week 4	Week 5	Week 6	Week 7	Week 8
wnVVO [arb. units]	Normal	44.16 ± 1.28	47.73 ± 1.42	46.60 ± 0.26	45.07 ± 0.89	43.47 ± 1.98	45.34 ± 0.89	43.38 ± 1.91	46.25 ± 0.92	44.68 ± 1.15
	SLD	44.24 ± 1.29	38.01 ± 1.61	37.41 ± 0.55	28.66 ± 1.19	26.71 ± 2.69	26.83 ± 1.51	29.72 ± 0.31	27.68 ± 0.62	28.41 ± 0.63
p-value	Norm. - SLD	1.000	0.029*	0.029*	0.029*	0.029*	0.029*	0.029*	0.029*	0.029*
wnFMBV [arb. units]	Normal	29.64 ± 1.63	32.03 ± 0.84	30.93 ± 0.39	31.65 ± 0.89	29.30 ± 1.22	31.86 ± 0.25	31.10 ± 1.60	32.88 ± 0.54	31.07 ± 0.16
	SLD	29.32 ± 0.91	25.06 ± 1.18	24.43 ± 0.58	18.50 ± 0.62	17.54 ± 1.64	17.02 ± 1.05	19.59 ± 0.28	17.98 ± 0.45	19.05 ± 0.26
p-value	Norm. - SLD	0.686	0.029*	0.029*	0.029*	0.029*	0.029*	0.029*	0.029*	0.029*
VBD [mm^{-3}]	Normal	4.12 ± 0.37	4.73 ± 0.37	4.48 ± 0.23	3.82 ± 0.06	4.01 ± 0.33	3.79 ± 0.18	4.01 ± 0.19	4.11 ± 0.38	4.20 ± 0.21
	SLD	4.38 ± 0.36	3.28 ± 0.24	3.31 ± 0.15	2.22 ± 0.41	1.87 ± 0.43	1.61 ± 0.33	1.98 ± 0.15	2.18 ± 0.06	1.65 ± 0.28
p-value	Norm. - SLD	0.686	0.029*	0.029*	0.029*	0.029*	0.029*	0.029*	0.029*	0.029*
SOAM [-]	Normal	8.57 ± 0.03	8.60 ± 0.01	8.56 ± 0.03	8.62 ± 0.02	8.58 ± 0.03	8.60 ± 0.05	8.51 ± 0.03	8.55 ± 0.06	8.57 ± 0.03
	SLD	8.50 ± 0.02	8.67 ± 0.04	8.67 ± 0.03	8.69 ± 0.08	8.72 ± 0.06	8.86 ± 0.02	8.86 ± 0.03	8.77 ± 0.03	8.86 ± 0.06
p-value	Norm. - SLD	0.057	0.686	0.114	0.686	0.057	0.029*	0.029*	0.057	0.029*
EAC [dB/cm/MHz]	Normal	0.82 ± 0.01	0.79 ± 0.03	0.81 ± 0.05	0.76 ± 0.07	0.66 ± 0.04	0.79 ± 0.05	0.82 ± 0.04	0.75 ± 0.03	0.77 ± 0.05
	SLD	0.80 ± 0.04	0.89 ± 0.02	0.86 ± 0.02	0.90 ± 0.05	0.95 ± 0.03	0.98 ± 0.03	1.09 ± 0.06	1.13 ± 0.05	1.27 ± 0.05
p-value	Norm. - SLD	0.886	0.057	1.000	0.200	0.029*	0.029*	0.029*	0.029*	0.029*
FDR [-]	Normal	0.63 ± 0.03	0.64 ± 0.03	0.62 ± 0.04	0.55 ± 0.04	0.61 ± 0.03	0.56 ± 0.04	0.58 ± 0.04	0.57 ± 0.04	0.63 ± 0.03
	SLD	0.72 ± 0.02	0.52 ± 0.01	0.45 ± 0.01	0.42 ± 0.03	0.43 ± 0.01	0.42 ± 0.02	0.45 ± 0.02	0.42 ± 0.01	0.40 ± 0.01
p-value	Norm. - SLD	0.114	0.029*	0.029*	0.114	0.029*	0.029*	0.057	0.029*	0.029*

Blood test	Group	Week 0	Week 1	Week 3	Week 5	Week 7	Week 8
ALT [U/L]	Normal	38.25 ± 2.02	41.50 ± 2.25	29.00 ± 3.72	16.25 ± 3.20	35.50 ± 1.94	33.00 ± 1.41
	SLD	41.25 ± 4.05	40.25 ± 9.94	133.75 ± 30.41	199.00 ± 49.49	124.25 ± 18.07	146.00 ± 40.51
p-value	Norm. - SLD	0.686	0.571	0.029*	0.029*	0.029*	0.029*
AST [U/L]	Normal	102.50 ± 16.32	73.25 ± 2.02	150.75 ± 50.19	60.00 ± 7.33	82.75 ± 15.98	88.75 ± 15.47
	SLD	79.00 ± 15.20	102.25 ± 23.69	184.00 ± 24.53	177.00 ± 25.06	172.50 ± 29.47	161.50 ± 24.02
p-value	Norm. - SLD	0.486	1.000	0.686	0.029*	0.343	0.057
TBIL [mg/dl]	Normal	0.20 ± 0.04	0.18 ± 0.03	0.20 ± 0.06	0.25 ± 0.03	0.38 ± 0.05	0.28 ± 0.05
	SLD	0.25 ± 0.03	0.25 ± 0.03	0.63 ± 0.05	0.65 ± 0.10	0.78 ± 0.13	0.88 ± 0.18
p-value	Norm. - SLD	0.657	0.286	0.029*	0.029*	0.171	0.029*

Supplementary Table 3. Histological quantification results of the SLD progression monitoring and validation experiments. Hepatic fat percentage was quantified from ORO staining, and microvascular damage was quantified by CD 31 and LYVE1 staining. Statistical significance was assessed using two-sided Mann–Whitney test without adjustment for multiple comparisons; *p < 0.05, **p < 0.01, ***p < 0.001.

Histological quantification	Normal week 0	SLD weeks 1–2	SLD weeks 3–4	SLD week 8	Normal week 8
Fat [%]	1.42 ± 0.44	32.88 ± 4.75	47.69 ± 2.65	68.01 ± 4.94	0.81 ± 0.11
p-value (vs Normal week 0)		0.0007***	0.0007***	0.016*	0.413
p-value (vs SLD weeks 1-2)			0.064	0.002**	0.002**
p-value (vs SLD weeks 3-4)				0.004**	0.002**
p-value (vs SLD week 8)					0.029*

Histological quantification	Normal week 0	SLD weeks 1–2	SLD weeks 3–4	SLD week 8	Normal week 8
CD31 density [%]	36.77 ± 1.10	34.85 ± 1.11	26.28 ± 1.03	22.67 ± 1.40	34.12 ± 1.12
p-value (vs Normal week 0)		0.513	0.0007***	0.016*	0.286
p-value (vs SLD weeks 1-2)			0.0004***	0.002**	0.839
p-value (vs SLD weeks 3-4)				0.076	0.002**
p-value (vs SLD week 8)					0.029*

Histological quantification	Normal week 0	SLD week 2	SLD week 4	SLD week 8	Normal week 8
LYVE1 density [%]	43.68 ± 2.31	30.61 ± 1.60	28.16 ± 0.61	29.88 ± 3.00	42.62 ± 1.28
p-value (vs Normal week 0)		0.029*	0.029*	0.029*	0.488
p-value (vs SLD week 2)			0.2	0.886	0.029*
p-value (vs SLD week 4)				0.886	0.029*
p-value (vs SLD week 8)					0.029*

Supplementary Table 4. Statistical analyses of the SLD recovery monitoring experiments. Quantitative US indices were monitored over 8 weeks. Weight-normalized vessel volume occupancy, wnVVO; weight-normalized fractional moving blood volume, wnFMBV; vessel bifurcation density, VBD; sum of angles metric, SOAM; estimated attenuation coefficient, EAC; and focal disturbance ratio, FDR. Blood biomarkers such as alanine aminotransferase (ALT), aspartate transaminase (AST), and total bilirubin (TBIL) were tested. Statistical significance was assessed using two-sided Mann–Whitney test without adjustment for multiple comparisons; *p < 0.05.

US index	Week 0	Week 1	Week 2	Week 3	Week 4	Week 5	Week 6	Week 7	Week 8
wnVVO [arb. units]	45.19 ± 0.71	38.63 ± 0.69	37.58 ± 1.78	32.80 ± 2.01	32.37 ± 1.67	34.83 ± 1.47	38.51 ± 1.32	40.46 ± 1.38	41.64 ± 0.51
p-value (vs Norm.)	0.686	0.029*	0.029*	0.029*	0.029*	0.029*	0.114	0.057	0.057
p-value (vs SLD.)	0.686	1.000	0.886	0.200	0.343	0.029*	0.029*	0.029*	0.029*
wnFMBV [arb. units]	30.45 ± 1.19	25.42 ± 0.22	24.52 ± 1.05	21.71 ± 1.13	21.55 ± 0.89	23.96 ± 1.27	27.67 ± 0.83	27.83 ± 0.65	28.57 ± 0.34
p-value (vs Norm.)	0.686	0.029*	0.029*	0.029*	0.029*	0.029*	0.200	0.029*	0.029*
p-value (vs SLD.)	0.686	0.486	1.000	0.114	0.114	0.029*	0.029*	0.029*	0.029*
VBD [mm^{-3}]	4.49 ± 0.20	4.03 ± 0.22	3.39 ± 0.33	2.61 ± 0.36	2.28 ± 0.27	2.32 ± 0.27	2.85 ± 0.30	3.68 ± 0.36	3.59 ± 0.25
p-value (vs Norm.)	0.486	0.343	0.057	0.029*	0.029*	0.029*	0.057	0.343	0.200
p-value (vs SLD.)	0.886	0.057	0.686	0.486	0.686	0.200	0.029*	0.029*	0.029*
SOAM [-]	8.58 ± 0.04	8.59 ± 0.03	8.74 ± 0.02	8.75 ± 0.08	8.76 ± 0.05	8.75 ± 0.04	8.70 ± 0.05	8.57 ± 0.04	8.61 ± 0.02
p-value (vs Norm.)	0.686	0.686	0.029*	0.343	0.057	0.029*	0.057	0.686	0.343
p-value (vs SLD.)	0.200	0.200	0.114	0.886	0.886	0.057	0.029*	0.057	0.029*
EAC [dB/cm/MHz]	0.77 ± 0.02	0.90 ± 0.03	0.84 ± 0.04	0.91 ± 0.03	0.94 ± 0.03	0.89 ± 0.06	0.89 ± 0.02	0.89 ± 0.03	0.85 ± 0.02
p-value (vs Norm.)	0.029*	0.057	0.686	0.114	0.029*	0.343	0.343	0.029*	0.686
p-value (vs SLD.)	0.343	0.686	0.686	0.686	0.886	0.343	0.029*	0.029*	0.029*
FDR [-]	0.68 ± 0.05	0.46 ± 0.03	0.52 ± 0.01	0.47 ± 0.03	0.44 ± 0.02	0.47 ± 0.01	0.50 ± 0.02	0.50 ± 0.02	0.52 ± 0.02
p-value (vs Norm.)	0.486	0.029*	0.057	0.200	0.029*	0.029*	0.200	0.200	0.057
p-value (vs SLD.)	0.686	0.057	0.029*	0.486	0.686	0.029*	0.114	0.029*	0.029*

Blood test	Week 0	Week 1	Week 3	Week 5	Week 7	Week 8
ALT [U/L]	40.50 ± 4.33	31.50 ± 2.72	125.75 ± 37.88	98.25 ± 27.72	33.75 ± 1.55	31.00 ± 3.11
p-value (vs Norm.)	0.686	0.057	0.029*	0.029*	0.657	1.000
p-value (vs SLD.)	1.000	0.771	1.000	0.114	0.029*	0.029*
AST [U/L]	72.25 ± 4.48	80.00 ± 8.03	173.25 ± 33.42	157.75 ± 34.81	79.75 ± 4.52	92.25 ± 11.87
p-value (vs Norm.)	0.286	0.314	0.686	0.029*	0.543	1.000
p-value (vs SLD.)	0.629	0.629	0.886	0.486	0.029*	0.114
TBIL [mg/dl]	0.23 ± 0.02	0.30 ± 0.06	0.53 ± 0.17	0.35 ± 0.05	0.25 ± 0.05	0.23 ± 0.06
p-value (vs Norm.)	1.000	0.286	0.171	0.286	0.114	0.657
p-value (vs SLD.)	1.000	0.657	0.371	0.057	0.029*	0.029*

Supplementary Table 5. Comparative histological quantification results between SLD recovery and SLD progression monitoring with validation experiments. Hepatic fat percentage was quantified from ORO staining, and microvascular damage was quantified CD 31 and LYVE1 staining. Statistical significance was assessed using two-sided Mann–Whitney test without adjustment for multiple comparisons; *p < 0.05, **p < 0.01.

Histological Quantification	Fat [%]	*p*-value (vs Norm. week 8)	*p*-value (vs SLD week 8)	*p*-value (vs week 0)	*p*-value (vs weeks 1–2)	*p*-value (vs weeks 3–4)
Recovery (at week 8)	5.57 ± 2.78	0.029*	0.029*	0.111	0.004**	0.002**

Histological Quantification	CD31 density [%]	*p*-value (vs Norm. week 8)	*p*-value (vs SLD week 8)	*p*-value (vs week 0)	*p*-value (vs weeks 1–2)	*p*-value (vs weeks 3–4)
Recovery (at week 8)	30.32 ± 1.44	0.114	0.029*	0.016*	0.076	0.036*

Histological Quantification	LYVE1 density [%]	*p*-value (vs Norm. week 8)	*p*-value (vs SLD week 8)	*p*-value (vs week 0)	*p*-value (vs weeks 1–2)	*p*-value (vs week 4)
Recovery (at week 8)	42.78 ± 1.02	1.000	0.029*	0.686	0.029*	0.029*

Supplementary Table 6. Combination order of all US indices.

Index	Combination
1	wnVVO
2	wnFMBV
3	VBD
4	SOAM
5	EAC
6	FDR
7	wnVVO + wnFMBV
8	wnVVO + VBD
9	wnVVO + SOAM
10	wnVVO + EAC
11	wnVVO + FDR
12	wnFMBV + VBD
13	wnFMBV + SOAM
14	wnFMBV + EAC
15	wnFMBV + FDR
16	VBD + SOAM
17	VBD + EAC
18	VBD + FDR
19	SOAM + EAC
20	SOAM + FDR
21	EAC + FDR
22	wnVVO + wnFMBV + VBD
23	wnVVO + wnFMBV + SOAM
24	wnVVO + wnFMBV + EAC
25	wnVVO + wnFMBV + FDR
26	wnVVO + VBD + SOAM
27	wnVVO + VBD + EAC
28	wnVVO + VBD + FDR
29	wnVVO + SOAM + EAC
30	wnVVO + SOAM + FDR
31	wnVVO + EAC + FDR
32	wnFMBV + VBD + SOAM
33	wnFMBV + VBD + EAC
34	wnFMBV + VBD + FDR
35	wnFMBV + SOAM + EAC
36	wnFMBV + SOAM + FDR
37	wnFMBV + EAC + FDR
38	VBD + SOAM + EAC
39	VBD + SOAM + FDR
40	VBD + EAC + FDR
41	SOAM + EAC + FDR
42	wnVVO + wnFMBV + VBD + SOAM
43	wnVVO + wnFMBV + VBD + EAC
44	wnVVO + wnFMBV + VBD + FDR
45	wnVVO + wnFMBV + SOAM + EAC
46	wnVVO + wnFMBV + SOAM + FDR
47	wnVVO + wnFMBV + EAC + FDR
48	wnVVO + VBD + SOAM + EAC
49	wnVVO + VBD + SOAM + FDR
50	wnVVO + VBD + EAC + FDR
51	wnVVO + SOAM + EAC + FDR
52	wnFMBV + VBD + SOAM + EAC
53	wnFMBV + VBD + SOAM + FDR
54	wnFMBV + VBD + EAC + FDR
55	wnFMBV + SOAM + EAC + FDR
56	VBD + SOAM + EAC + FDR
57	wnVVO + wnFMBV + VBD + SOAM + EAC
58	wnVVO + wnFMBV + VBD + SOAM + FDR
59	wnVVO + wnFMBV + VBD + EAC + FDR
60	wnVVO + wnFMBV + SOAM + EAC + FDR
61	wnVVO + VBD + SOAM + EAC + FDR
62	wnFMBV + VBD + SOAM + EAC + FDR
63	wnVVO + wnFMBV + VBD + SOAM + EAC + FDR

References

1. Ng A, Swanevelder J. Resolution in ultrasound imaging. Continuing Education in Anaesthesia, Critical Care & Pain. 2011;11(5):186-92.
2. Pinkert MA, Hall TJ, Eliceiri KW. Challenges of conducting quantitative ultrasound with a multimodal optical imaging system. Physics in Medicine & Biology. 2021;66(3):035008.
3. Faul F, Erdfelder E, Lang A-G, Buchner A. G* Power 3: A flexible statistical power analysis program for the social, behavioral, and biomedical sciences. Behavior research methods. 2007;39(2):175-91.
4. Faul F, Erdfelder E, Buchner A, Lang A-G. Statistical power analyses using G* Power 3.1: Tests for correlation and regression analyses. Behavior research methods. 2009;41(4):1149-60.
5. Choi C, Choi W, Kim J, Kim C. Non-invasive photothermal strain imaging of non-alcoholic fatty liver disease in live animals. IEEE Transactions on Medical Imaging. 2021;40(9):2487-95.
