# Supplementary material for: 3D multiparametric ultrasound imaging of steatotic liver disease in a study with male rats
Source: Nat Commun. 2025 Nov 20;16:10226. doi: 10.1038/s41467-025-65046-x (PMC12635210; doi:10.1038/s41467-025-65046-x)
Supplement: Supplementary file 2 — Description of additional supplementary files [file 41467_2025_65046_MOESM2_ESM.pdf]

Description of Additional Supplementary Files

Supplementary Video 1. Real-time respiratory triggering and ensemble selection.

This video shows the acquisition of US frames corresponding to cardiac cycles using real-time B-mode US–based respiratory triggering, followed by the selection of ensemble frames for UFD processing.

Supplementary Video 2. 3D hepatic USI sequence, liver region segmentation, and quantitative 3D PD through vascular modeling.

This video demonstrates the generation of the final vessel-segmented 3D PD from acquired volumetric US data.
