# Supplementary material for: 3D multiparametric ultrasound imaging of steatotic liver disease in a study with male rats
Source: Nat Commun. 2025 Nov 20;16:10226. doi: 10.1038/s41467-025-65046-x (PMC12635210; doi:10.1038/s41467-025-65046-x)
Supplement: Supplementary file 5 — Reporting Summary [file 41467_2025_65046_MOESM5_ESM.pdf]

nature portfolio

Corresponding author(s): Chulhong Kim

Last updated by author(s): Sep 14, 2025

Reporting Summary

Nature Portfolio wishes to improve the reproducibility of the work that we publish. This form provides structure for consistency and transparency in reporting. For further information on Nature Portfolio policies, see our Editorial Policies and the Editorial Policy Checklist.

Statistics

For all statistical analyses, confirm that the following items are present in the figure legend, table legend, main text, or Methods section.

n/a	Confirmed	
☐	☒	The exact sample size (*n*) for each experimental group/condition, given as a discrete number and unit of measurement
☐	☒	A statement on whether measurements were taken from distinct samples or whether the same sample was measured repeatedly
☐	☒	The statistical test(s) used AND whether they are one- or two-sided<br>*Only common tests should be described solely by name; describe more complex techniques in the Methods section.*
☐	☒	A description of all covariates tested
☐	☒	A description of any assumptions or corrections, such as tests of normality and adjustment for multiple comparisons
☐	☒	A full description of the statistical parameters including central tendency (e.g. means) or other basic estimates (e.g. regression coefficient) AND variation (e.g. standard deviation) or associated estimates of uncertainty (e.g. confidence intervals)
☐	☒	For null hypothesis testing, the test statistic (e.g. *F*, *t*, *r*) with confidence intervals, effect sizes, degrees of freedom and *P* value noted<br>*Give P values as exact values whenever suitable.*
☒	☐	For Bayesian analysis, information on the choice of priors and Markov chain Monte Carlo settings
☒	☐	For hierarchical and complex designs, identification of the appropriate level for tests and full reporting of outcomes
☐	☒	Estimates of effect sizes (e.g. Cohen's *d*, Pearson's *r*), indicating how they were calculated

Our web collection on statistics for biologists contains articles on many of the points above.

Software and code

Policy information about availability of computer code

Data collection	Experimental data were collected using the Vantage 256 system (Verasonics Inc.) implemented within the MATLAB R2022a framework (MathWorks, Inc.).
Data analysis	MATLAB R2022b and R2024a (MathWorks, Inc.) were utilized. Data and image processing are described in detail in the Methods section. The open-source OBNLM package (https://www.bic.mni.mcgill.ca/PersonalCoupepierrick/OBNLMFilter) and the Jerman filter (https://github.com/timjerman/JermanEnhancementFilter) were employed.

For manuscripts utilizing custom algorithms or software that are central to the research but not yet described in published literature, software must be made available to editors and reviewers. We strongly encourage code deposition in a community repository (e.g. GitHub). See the Nature Portfolio guidelines for submitting code & software for further information.

Data

Policy information about availability of data

All manuscripts must include a data availability statement. This statement should provide the following information, where applicable:

- Accession codes, unique identifiers, or web links for publicly available datasets
- A description of any restrictions on data availability
- For clinical datasets or third party data, please ensure that the statement adheres to our policy

The main data supporting the findings of this study are included in the main text, figures, and supplementary information. The representative subset of the US raw

data generated in this study has been deposited in the Zenodo database [https://doi.org/ 10.5281/zenodo.16918233]. Due to the large size of the entire raw dataset (~50 TB), only the subset is hosted on Zenodo; however, the complete dataset can be provided upon request to the corresponding author. Source data are provided in this paper.

Research involving human participants, their data, or biological material

Policy information about studies with human participants or human data. See also policy information about sex, gender (identity/presentation), and sexual orientation and race, ethnicity and racism.

Reporting on sex and gender	Our human liver imaging was conducted as a preliminary imaging; therefore, volunteers were recruited regardless of sex, and sex-based analyses were not performed.
Reporting on race, ethnicity, or other socially relevant groupings	Imaging was conducted at POSTECH (Republic of Korea), where the researcher is based, and involved Korean participants.
Population characteristics	Population characteristics were not considered, as this was preliminary imaging.
Recruitment	A single volunteer was recruited randomly, and no compensation was provided.
Ethics oversight	Human volunteer experiments were performed in accordance with a protocol approved by the Institutional Review Board of Pohang University of Science and Technology (POSTECH-PIRB-2023-A001-C2 , approved on 17 Jan. 2025).

Note that full information on the approval of the study protocol must also be provided in the manuscript.

Field-specific reporting

Please select the one below that is the best fit for your research. If you are not sure, read the appropriate sections before making your selection.

☒ Life sciences ☐ Behavioural & social sciences ☐ Ecological, evolutionary & environmental sciences

For a reference copy of the document with all sections, see nature.com/documents/nr-reporting-summary-flat.pdf

Life sciences study design

All studies must disclose on these points even when the disclosure is negative.

Sample size	The total sample size (n = 37) was calculated using G*Power based on the results from previous study. Detailed descriptions are provided in the Supplementary Note.
Data exclusions	No data were excluded in this study.
Replication	The results for each index were derived only once from each subject (i.e., biological replicate). Our approach demonstrated excellent inter-subject and inter-system reproducibility, as described in the Discussion section, with the supporting data provided in the Supplementary Information. We have enabled successful long-term volumetric scan observations by optimizing the volume scanning process through real-time US-based respiratory gating and by strategically applying various techniques to establish a stable vascular modeling framework, thereby enhancing imaging reproducibility.
Randomization	All animals were randomly allocated to each group in accordance with the experimental design: (1) an SLD progression monitoring scheme involving normal rats (n = 4) and SLD-conditioned rats (n = 4), (2) an SLD validation scheme using 25 rats divided into five groups (n = 5 per group) based on induced-SLD durations of 0, 1, 2, 3, and 4 weeks, and (3) an SLD recovery monitoring scheme involving recovery-conditioned rats (n = 4).
Blinding	Group allocation was performed in a blinded manner; however, complete blinding during data collection was not feasible, as SLD-conditioned rats required continuous feeding with the MCD diet.

Reporting for specific materials, systems and methods

We require information from authors about some types of materials, experimental systems and methods used in many studies. Here, indicate whether each material, system or method listed is relevant to your study. If you are not sure if a list item applies to your research, read the appropriate section before selecting a response.

Materials & experimental systems

n/a	Involved in the study	
☐	☒	Antibodies
☒	☐	Eukaryotic cell lines
☒	☐	Palaeontology and archaeology
☐	☒	Animals and other organisms
☒	☐	Clinical data
☒	☐	Dual use research of concern
☒	☐	Plants

Methods

n/a	Involved in the study	
☒	☐	ChIP-seq
☒	☐	Flow cytometry
☒	☐	MRI-based neuroimaging

Antibodies

Antibodies used	We used the primary antibodies anti-CD31 (1:200, AF3628, R&D) and anti-LYVE-1 (1:200, AF7939, R&D), followed by the secondary antibodies rabbit anti-goat Alexa Fluor 488 (1:2000, Invitrogen) and donkey anti-sheep Alexa Fluor 488 (1:2000, Jackson ImmunoResearch).
Validation	CD31 (AF3628, R&D Systems): rat, immunofluorescence, https://www.rndsystems.com/products/human-mouse-rat-cd31-pecam-1-antibody_af3628.<br>LYVE1 (AF7939, R&D Systems): rat, immunofluorescence, https://www.rndsystems.com/products/rat-lyve-1-antibody_af7939.<br>Both antibodies have been validated by the manufacturer and in previous studies for use in rat tissue and immunofluorescence applications.

Animals and other research organisms

Policy information about studies involving animals; ARRIVE guidelines recommended for reporting animal research, and Sex and Gender in Research

Laboratory animals	A total of 37 male Wistar rats (Rattus norvegicus, outbred, CrljOri:Wistar, 7 weeks old, approximately 200g at week 0), obtained from Orient Bio (Seongnam, Korea), were involved in our study. The animals were raised for varying durations, ranging from 0 to 8 weeks.
Wild animals	*Provide details on animals observed in or captured in the field; report species and age where possible. Describe how animals were caught and transported and what happened to captive animals after the study (if killed, explain why and describe method; if released, say where and when) OR state that the study did not involve wild animals.*
Reporting on sex	Male Wistar rats were used in this study, as a previous study demonstrated that male Wistar rats exhibit the most pronounced steatosis in response to the MCD diet. Sex-based analyses were not conducted, as only male animals were included in the study.
Field-collected samples	*For laboratory work with field-collected samples, describe all relevant parameters such as housing, maintenance, temperature, photoperiod and end-of-experiment protocol OR state that the study did not involve samples collected from the field.*
Ethics oversight	All procedures for experimental animal were reviewed by the Institutional Animal Care and Use Committee of Pohang University of Science and Technology (POSTECH-2023-0099, approved on 21 Aug. 2023) and were strictly followed.

Note that full information on the approval of the study protocol must also be provided in the manuscript.

Plants

Seed stocks	*Report on the source of all seed stocks or other plant material used. If applicable, state the seed stock centre and catalogue number. If plant specimens were collected from the field, describe the collection location, date and sampling procedures.*
Novel plant genotypes	*Describe the methods by which all novel plant genotypes were produced. This includes those generated by transgenic approaches, gene editing, chemical/radiation-based mutagenesis and hybridization. For transgenic lines, describe the transformation method, the number of independent lines analyzed and the generation upon which experiments were performed. For gene-edited lines, describe the editor used, the endogenous sequence targeted for editing, the targeting guide RNA sequence (if applicable) and how the editor was applied.*
Authentication	*Describe any authentication procedures for each seed stock used or novel genotype generated. Describe any experiments used to assess the effect of a mutation and, where applicable, how potential secondary effects (e.g. second site T-DNA insertions, mosiacism, off-target gene editing) were examined.*
